# Cultural Adaptation of a Web-Based Ostomy Care Intervention for Hispanic Patients With Cancer and Caregivers: Mixed Methods Study

**DOI:** 10.2196/70354

**Published:** 2026-02-03

**Authors:** Xiaomeng Wang, Lixin Song, Amy Vondenberger, Fei Yu, Jia Liu, Roxana Delgado, Robert Svatek, Natalia Villegas Rodriguez, Lurheinna Rosado Rivera, Mark Bonnen

**Affiliations:** 1 School of Nursing, The University of Texas Health Science Center at San Antonio, San Antonio, TX, United States; 2 School of Nursing, Mays Cancer Center, The University of Texas Health Science Center at San Antonio, 7703 Floyd Curl Dr, San Antonio, TX, 78229, United States, 1 210 450 8561; 3 School of Information and Library Science, University of North Carolina at Chapel Hill, Chapel Hill, NC, United States; 4 Department of Urology, Long School of Medicine, The University of Texas Health Science Center at San Antonio, San Antonio, TX, United States; 5 Mays Cancer Center, The University of Texas Health Science Center, San Antonio, San Antonio, TX, United States; 6 School of Nursing, University of North Carolina at Chapel Hill, Chapel Hill, NC, United States; 7 Department of Radiation Oncology, Long School of Medicine, The University of Texas Health Science Center at San Antonio, San Antonio, TX, United States

**Keywords:** ostomy, Hispanic, eHealth, digital platform, caregiver, patient with cancer, cultural adaptation, persuasive technology.

## Abstract

**Background:**

Ostomy creation for cancer treatment negatively impacts the quality of life of both patients and caregivers. Hispanic patients with cancer and caregivers often face additional challenges, including limited access to supportive care programs.

**Objective:**

This study aimed to examine the experiences and preferences of Hispanic patients with cancer living with ostomies and their caregivers to inform the cultural adaptation of an existing intervention program and the design of Ostomy Self-Care Program (Programa de AutoCuidado de Estoma [PACE]).

**Methods:**

In this 2-stage study, conducted between March and August 2023 in San Antonio, Texas, we used a qualitatively driven mixed methods design, starting with an initial survey followed by qualitative interviews to explore the experiences, needs, and intervention preferences of Hispanic patients and caregivers managing ostomy care. We used Braun and Clarke’s 6-phase thematic analysis approach to analyze the qualitative data and performed descriptive analysis for the quantitative data. Subsequently, we applied affinity diagramming and persuasive systems design principles to guide the design of PACE.

**Results:**

In total, 14 Hispanic participants managing an ostomy (9 patients with cancer and 5 caregivers) completed a survey and participated in interviews, continuing until data saturation was reached. Participants had a mean age of 58.9 (SD 13.01, range 37‐79) years, and most (n=12) reported a high school diploma or General Educational Development as their highest education level. Around 5 (36%) participants scored below 26 on the eHealth Literacy Scale (eHEALS), indicating low digital health literacy, and the average Charlson Comorbidity Index (CCI) was 3.21 (SD 1.86, range 0‐6). Overall, 3 major themes emerged from the qualitative data analysis, namely perceptions of living with an ostomy, seeking support, and postsurgery challenges. Additionally, two primary themes emerged from participant interviews: (1) importance of preferred language and multimedia delivery and (2) patients and caregivers desire early introduction, multimodal delivery of materials, and inclusion of peer and family support. These themes informed the design and development of a culturally appropriate, web-based, bilingual PACE intervention that integrates content visualization, cultural adaptations, and persuasive technologies—strategies designed to encourage user engagement.

**Conclusions:**

Our findings emphasize the importance of understanding the ostomy care experiences, supportive care needs, and intervention preferences of Hispanic patients and caregivers. Informed by stakeholders’ insights, we culturally adapted the original intervention program using persuasive systems design principles to design and develop the PACE intervention, aiming to enhance engagement among Hispanic patients with cancer and caregivers, support effective self-management of ostomy care, and improve health outcomes.

## Introduction

Ostomy creation can be performed as part of cancer treatment for bladder, colorectal, cervical, ovarian, and uterine cancers. The surgery involves creating an opening on the anterior abdominal wall to allow defecation or urination [1]. This procedure can complicate the patient’s ability to resume normal daily activities and further impact quality of life (QOL) [2]. While supportive care and management can sustain QOL after ostomy creation, a gap remains in available care resources that are tailored to minorities, including Hispanic patients with cancer and caregivers.

Compared to their non-Hispanic counterparts, Hispanic patients with cancer and their caregivers in the United States face additional challenges throughout cancer survivorship. These barriers include socioeconomic difficulties [3], limited access to health care [4], language barriers, and financial strain [5]. Additionally, this population experiences a higher rate of being uninsured [6]. Approximately 51.3% of Hispanics in the United States need translation services during medical consultations [7]. These language barriers can hinder effective communication and potentially lead to misunderstanding home care instructions [8]. Patients undergoing an ostomy creation surgery typically use more health care resources [9], which poses a further detriment to the Hispanic population, as they often face significant challenges in obtaining health care resources[10]. In addition, cultural beliefs and practices play a crucial role in illness management. In Hispanic culture, families traditionally take on the responsibility of caring for their sick members [11], and they tend to be more involved in ostomy care than any other medical or nursing tasks [12]. A study shows that involving family members in group training sessions can significantly improve outcomes among Hispanic patients and families[13]. Recognizing the needs of both Hispanic patients and their families is essential for providing adequate support and intervention in postostomy care.

Web-based interventions offer a promising way to support self-management for patients with chronic conditions and their families [14]. Research suggests that, compared to non–web-based interventions, these web-based interventions are effective in promoting knowledge acquisition and behavioral change [15]. As a cost-effective solution, they can particularly benefit underserved groups, such as Hispanic communities [16], through overcoming barriers to health care access. Offering content in Spanish further addresses language barriers and increases access [17]. Additionally, it provides a convenient way for patients to share information within their community, fostering peer support among people who have similar experiences [18].

As part of our preparatory work to address the care needs of Hispanic patients with cancer and caregivers, our multidisciplinary team previously translated the PRISMS (Patient-Reported Outcomes-Informed Symptom Management System) [19] into Spanish. PRISMS is an eHealth intervention that integrates a program with wearable devices to support patients with cancer and their caregivers during the postostomy care transition from hospital to at-home self-management. PRISMS aims to reduce preventable emergency room use and readmissions [19]. To ensure the cultural appropriateness of the translated materials, we engaged 10 bilingual translators, including clinical nurses, nursing students, and faculty, from Chile, Mexico, Puerto Rico, Argentina, and Colombia. These bilingual, native Spanish speakers translated the English version of PRISMS into Spanish to accommodate various dialects while sensitively addressing cultural nuances.

Building on previous work, our current study explored the experiences of Hispanic patients with cancer and caregivers regarding ostomy care, as well as their preferences for ostomy care interventions, to inform cultural adaptation of PRISMS-Spanish using a mixed methods approach. We presented the PRISMS-Spanish materials to participants to facilitate discussion and gather their feedback to help inform our design of a web-based ostomy care intervention program—Programa de AutoCuidado de Estoma (PACE)—tailored for this specific underserved population. While some references cited in this paper use terms, such as “Latino/Latina,” we use “Hispanic” consistently throughout this study to align with the terminology used in participant recruitment.

## Methods

### Study Design Overview

In stage 1, we used a qualitatively driven mixed methods design with sequential data collection and parallel analysis. First, we made an initial phone call to each participant and collected quantitative data. Then we scheduled a Zoom (Zoom Communications) interview with each of them to collect qualitative data, which was typically within 1 month after the initial phone contact. The survey data provided a clear understanding of the characteristics, health literacy, and long-term mortality risk of Hispanic patients with cancer and caregivers. In contrast, the interview data and thematic analysis offered in-depth insights into their ostomy care experiences and preferences for intervention features and functions. We used Braun and Clarke’s 6-phase thematic analysis approach [20] to guide our data analysis.

In stage 2, we used affinity diagramming and the Persuasive Systems Design (PSD) model developed by Oinas-Kukkonen and Harjumaa [21] to guide PACE program design for effective user engagement [22,23].

### Stage 1: Exploration of Ostomy Care Experiences and Intervention Preference

We aimed to understand the experiences and information needs of Hispanic patients and caregivers. Additionally, we gathered intervention preferences based on participant feedback on PRISMS-Spanish materials.

#### Participants

Participants were recruited from the Mays Cancer Center in San Antonio, Texas, the seventh largest city in the United States, with a 65% Hispanic majority population [24]. Patients were eligible if they (1) were Hispanic adults with one of three types of ostomies (colostomy, ileostomy, or urostomy) for the treatment of bladder, colorectal, cervical, ovarian, or uterine cancer; (2) could read and speak Spanish; and (3) passed a cognitive assessment using the Mini-Mental State Examination [25]. Caregivers were included if they were adults serving as the patient’s primary caregiver and could read and speak Spanish. Eligible participants were contacted via phone to confirm their willingness to participate and to address any questions.

#### Data Collection

Quantitative and qualitative data were collected through a survey administered during an initial phone call, followed by a Zoom interview that began with a presentation of the PRISMS-Spanish materials. Each participant encounter was conducted in either English or Spanish, based on their language preference. The survey questionnaires, interview guides, and intervention materials were translated into Spanish by a certified translator and reviewed by bilingual research staff and nursing students.

The trained bilingual research staff and research assistant (AV and LRR) conducted the initial phone survey before the interview in a secure, private office space at the University of Texas Health Science Center at San Antonio (UTHSA). They read each survey question aloud and recorded participants’ responses into REDCap (Research Electronic Data Capture; Vanderbilt University) hosted by the UTHSA. The survey items included demographic questions, type of ostomy, cancer diagnosis, Charlson Comorbidity Index (CCI) [26], and the Electronic Health Literacy Scale (eHEALS) [27]. Participants were asked to complete the CCI and the eHEALS questionnaires to measure comorbidities and digital health literacy, which were critical for chronic illness management and for informing the PACE design. The CCI score is the summation of the number of comorbidities that participants reported. The eHEALS score, ranging from 8 to 40, assessed participants’ digital literacy, including knowledge, comfort level, and perceived abilities in using electronic health information, with scores below 26 indicating low health literacy.

Next, participants were scheduled for a Zoom interview session at their convenience. During the session, translated PRISMS-Spanish materials were first presented, either via screen share or by email if needed, followed by open-ended questions. The semistructured interview guide was collaboratively developed by the research team, informed by insights from the PRISMS study [19] and a review of existing interventions related to ostomy care, digital health literacy, and culturally tailored programs for Hispanic populations. Interview questions focused on four main areas: (1) experience of ostomy care, (2) feedback on PRISMS-Spanish materials, (3) preferences for intervention and delivery, and (4) preferred implementation strategies. Interview sessions lasted approximately 45‐60 minutes and were conducted between March and August 2023 by the research staff. All interviews were audio-recorded and transcribed verbatim via Zoom and reviewed by the project coordinator and trained research staff who had prior experience working with Spanish-speaking patients. We determined that data saturation was achieved when no new codes or themes emerged across interviews. This approach aligns with prior research indicating that qualitative data saturation often occurs within the first 12 interviews, with key themes emerging as early as 6 [28].

#### Data Analysis

Quantitative data from the initial phone surveys were exported from REDCap into Microsoft Excel and RStudio (Posit; version 4.4.1) for analysis. We performed descriptive statistics, including means, ranges, frequencies, SDs, and percentages, to summarize participant demographics, CCI scores, and eHEALS (Table 1). These analyses provided an overview of the sample’s characteristics and informed the interpretation of qualitative findings.

**Table 1.** Participant characteristics.

Variable	Patients (n=9), n	Caregivers (n=5), n	Both (n=14), n
Sex			
Male	4	2	6
Female	5	3	8
Race			
White	6	4	10
Other	3	1	4
Education			
<=7th-8th Grade	2	0	2
12th Grade	6	4	10
Bachelor’s degree	1	1	2
Employment status			
Work	3	2	5
Retired, age/choice	2	3	5
Disability/illness	3	0	3
Unable to work/perform normal activities	1	0	1
Marital status			
Single/never married	1	0	1
Married/domestic partner Partner	8	4	12
Long-term partner	0	1	1
Ostomy type			
Urostomy	2	3	5
Ileostomy	3	0	3
Colostomy	4	2	6
Age (years)			
30‐39	2	0	2
40‐49	0	1	1
50‐59	3	1	4
60‐69	2	2	4
>70	2	1	3
CCI^a^			
0	1	0	1
1-2	3	1	4
3-4	3	3	6
≥5	2	1	3
eHEALS score^b^			
<26	4	1	5
>26	5	4	9

a CCI: Charlson Comorbidity Index.

b eHEALS: eHealth Literacy Scale.

The deidentified qualitative interview data were analyzed using Braun and Clarke’s 6-phase approach to thematic analysis [20]. This approach was applied to ensure a credible analysis procedure and continued until no new themes were identified. The approach is detailed as follows:

(1) Familiarization: Two researchers (AV and LRR) independently read the interview transcripts and noted initial ideas and patterns. This process allowed researchers to gain an in-depth understanding of the participants’ overall experiences and needs.

(2) Generating initial codes: AV and LRR generated initial codes systematically across the entire dataset, using NVivo (Lumivero) qualitative data analysis software (version 10.2.1) and a preliminary codebook based on interview topics.

(3) Searching for themes: the research team collated the initial codes into broader themes by grouping related codes to generate potential themes to capture participants’ experiences.

(4) Reviewing themes: Team meetings were held to review themes and resolve discrepancies in theme identification, aiming for a minimum coding consistency agreement of 80% [29].

(5) Defining and naming themes: 3 researchers (AV, LRR, and XW) collaboratively defined and named each theme, ensuring that each theme was distinct and related to the aim of this study. Detailed descriptions were developed for each theme, supported by illustrative quotes from participants.

(6) Producing the report: Finally, the team synthesized the findings into a cohesive report, which was used to inform the cultural adaptation of PRISMS-Spanish and the design of PACE.

Although quantitative and qualitative data were collected sequentially, analysis was conducted in parallel. Quantitative descriptive statistics (eg, demographics, CCI, and eHEALS) were reviewed with qualitative codes and themes side by side. The integration occurred during the interpretation phase, where quantitative results were used to contextualize and support qualitative findings. The integrated results were synthesized into joint displays (Tables 2 and 3) to inform the design of the PACE intervention.

**Table 2.** Hispanic patients’ and caregivers’ experiences with ostomy care.

Themes and subthemes	Participant quotes
Ostomy perception	
Emotion	“Because it is kind of a shocking thing to see your internal organs hanging out of your body even though it’s a little bit, it’s still a hole in your belly and you got your guts or hanging out.” [Female, ileostomy, patient, eHEALS^a^ score=35, CCI^b^=2] “All the ugly stuff that nobody wants to show.” [Female, ileostomy, patient, eHEALS score=32, CCI=3]
Necessity	“And as well as many other people, it definitely has changed my perception to something that is scary to something that is a necessity at times if needed.” [Female, colostomy, patient, eHEALS score=38, CCI=4] “Knowing that I had it and I needed it to save my life.” [Female, colostomy, patient, eHEALS score=38, CCI=4]
Support	
Health care provider	“But I don’t feel that knowledge is there for a lot of health care workers.” [Female, colostomy, patient, eHEALS score=38, CCI=4]
Family and caregiver	“So I really depended on him (husband) to help me put a new one back on.” [Female, colostomy, patient, eHEALS score=38, CCI=4] “… but what really helped me a lot was her (wife) and my family that was a hundred percent behind me and you know they, gave me the faith because I didn’t have the faith at first.” [Male, colostomy, patient, eHEALS score=23, CCI=6] “I have an alternative. I do my husband’s changes for his urostomy bag. But my son also knows how to provide the care because his wife has an ostomy bag. So in a pinch if I’m not available, he can substitute for me.” [Female, urostomy, caregiver, eHEALS score=32, CCI=4] “So they supported me through the cancer, but they weren’t really sure how to help me or how to feel about it because it deals with stool and . I know they were really like kind of just supporting but not really familiar with it.” [Female, colostomy, patient, eHEALS score=38, CCI=4] “I think so, that they also involve families in these talks for them to also see how family members and the sick should be treated” [Male, colostomy, caregiver, eHEALS score=29, CCI=3]
Peer support	“I had a lot more difficulties with my ostomy then a lot of people but you know, I ended up going to peers that worked with an ostomy as opposed to the ostomy care nurses that they were giving me because they too still weren’t familiar with how to take care of my problem.” [Female, colostomy, patient, eHEALS score=38, CCI=4]
Self-care	“… it depends on what the circumstances are, but I think that the patient should do as much as possible to help themselves.” [Female, ileostomy, patient, eHEALS score=35, CCI=2]
Challenges	
Radiation side effects	“I couldn’t even lift up my arms after radiation ... if they have the ostomy bag for some reason, if they’re also undergoing radiation. Yeah, that’s bad.” [Female ileostomy, patient, eHEALS score=35, CCI=2]
Chemotherapy side effects	“here’s added things so sometimes chemos depending on which one some can cause a lot of diarrheas. Some can cause constipation.” [Female, colostomy, patient, eHEALS score=38, CCI=4]
Skin irritation	“You know, a lot of times people see, okay, it’s just, it’s just a little rash or whatever But actually, it’s maybe an infection or something else and they see it as it just being a little rash when it’s really not, you know.” [Male, ileostomy, patient, eHEALS score=34, CCI=0] “The skin irritation just lasted the entire time.” [Female, ileostomy, patient, eHEALS score=35, CCI=2] “Once in a while, he’ll have a little bit of irritation, but I’ll just clean it.” [Female, urostomy, caregiver, eHEALS score=32, CCI=4]
Leakage	“One reason was because, you know, I was having like a leak. And we’re like, well maybe, what are we doing wrong?” [Male, ileostomy, patient, eHEALS score=34, CCI=0] “Because I’m a heavier-set girl and obesity is rampant in the Latin community. My ostomy was retracted in so it was not sticking out like normal people.it had a lot more problems with leaking because it couldn’t get a good seal around the stoma.” [Female, colostomy, patient, eHEALS score=38, CCI=4]
Self-isolation	“I feel in the house we had everything organized and everything there for us to do the job, but taking it out of the houses where it was a little harder for me.” [Female, colostomy, patient, eHEALS score=38, CCI=4]
Caregiver stress and emotion	“I’m gonna say like a depression myself, but I kept it to myself.” [Female, colostomy, caregiver, eHEALS score=19, CCI=1] “We didn’t know what else to do. but you know at that point my dad had lost a lot of blood.” [Male, urostomy, eHEALS score=34, CCI=4]

a CCI: Charlson Comorbidity Index.

b eHEALS: eHealth Literacy Scale.

**Table 3.** Preferences for the ostomy care intervention among Hispanic patients and caregivers.

Themes and subthemes	Participant quotes
Importance of preferred language and multimedia delivery	
Content and delivery format	“Okay, the information is really, really helpful.” [Female urostomy, caregiver, eHEALS^a^ score=32, CCI^b^=4] “The YouTube (videos) really does help out a lot.” [Male, ileostomy, patient, eHEALS score=34, CCI=0] “You have some really good tips like though there was one that we were told about and it really had helped me now that I when I apply the bag on my husband.” [Female, urostomy, caregiver, eHEALS score=32, CCI=4]
Language	“… for one who does not speak English. There is nothing, there is nothing clearer than Spanish, so the explanation is excellent, it is very good.” [Female, colostomy, patient, eHEALS score=25, CCI=1]
Need for early, multimodal materials and peer and family support	
Delivery platform	“I do on a phone and do a desktop.” [Female, urostomy, patient, eHEALS score=40, CCI=6] “For me it was more telephone because that’s what I used more as far as online and researching stuff.” [Female, ileostomy, patient, eHEALS score=32, CCI=3] “I happen to like computers, … but I think not a lot of people, especially if they’re older ... So he’s not computer savvy either. So I think maybe like a handout or in a notebook kind of form would be better. With pictures.” [Female, urostomy, patient, eHEALS=40, CCI=6]
Delivery timing	“This should be shown to the patients before they leave the hospital because they might not have the support once they get home.” [Female, urostomy, caregiver, eHEALS score=32, CCI=4] “Like before you get going home.” [Female, urostomy, caregiver, eHEALS score=32, CCI=4] “I think it’d be better if you were to be able to give them all the information ahead of time.” [Male, ileostomy, patient, eHEALS score=34, CCI=0]
Family and peer support	“We definitely needed help. So I definitely think helping caregivers or showing caregivers how to help is really important.” [Female, colostomy, Patient, eHEALS score=38, CCI=4] “I think it’s very important that anybody in the family that’s willing to be involved that they would get involved in case the person that normally does it for you or helps you with it is not available for whatever reason. I think other family members should be involved also.” [Male, urostomy, patient, eHEALS score=33, CCI=3] “I think it would be wise and I think it really helps if you ask somebody, somebody personal, somebody that’s gone through it.” [Male, ileostomy, patient, eHEALS score=34, CCI=0]

a CCI: Charlson Comorbidity Index.

b eHEALS: eHealth Literacy Scale.

### Stage 2: PACE Web-Based Intervention Program Design

#### Overview

Findings from stage 1 informed the cultural adaptation of PRISMS-Spanish and the design of the PACE prototype. We used affinity diagramming to organize the intervention contents and materials, and the PSD model to guide the design of the PACE program. Our multidisciplinary team, including digital health researchers, nursing faculty, user interface (UI) designers, web developers, informatics specialists, and clinicians from UTHSA and the University of North Carolina at Chapel Hill, held weekly hybrid meetings (in-person and via Zoom) over 6 months (from September 2023 to March 2024). Design decisions were made collaboratively during structured sessions focused on usability, cultural relevance, and visual accessibility, during which wireframes and prototypes were reviewed. Ideas and comments were synthesized and iteratively incorporated into the prototypes using affinity diagramming on a physical whiteboard, guided by the PSD model. A project coordinator documented meeting notes and key design decisions throughout the process.

#### Affinity Diagramming

We used affinity diagramming [30] to organize ostomy care materials across various web pages based on the content interrelationships to delineate the tasks and features of the new PACE program. Affinity diagramming is a commonly used user experience design approach that helps organize design ideas systematically [30]. Based on PRISMS-Spanish, the results from stage 1, and the affinity diagram, we generated 5 different types of prototypes for PACE by collaborating with a UI design class. Our multidisciplinary team analyzed and evaluated each prototype. Key features, such as website layout, visual and graphical representations, and navigation options were finalized to ensure the program would meet the needs of patients and caregivers.

#### Persuasive Systems Design

To ensure the appropriate application of these persuasive principles, we incorporated expert evaluation during the design process. We used PSD to guide the design of the culturally adapted PACE to optimize user engagement in the web-based intervention [31]. The PSD model includes 28 design principles across 4 categories (Textbox 1). Experts in health care, UI design, and health informatics provided feedback on the integration between the PSD model and PACE.

Textbox 1. **Persuasive system design model.**

**Categories of persuasive techniques and system design principles**

- Primary task support: reduction, tunneling, tailoring, personalization, self-monitoring, simulation, and rehearsal.
- Dialogue support: praise, rewards, reminders, suggestion, similarity, liking, and social role.
- Credibility support: trustworthiness, expertise, surface credibility, real-world feel, authority, third-party endorsements, and verifiability.
- Social support: social learning, social comparison, normative influence, social facilitation, cooperation, competition, and recognition.

### Ethical Considerations

This study was approved by the University of Texas Health San Antonio’s Institutional Review Board (IRB; number 20220693EX). Referred patients were contacted via phone to confirm their eligibility and willingness to participate and to answer their questions. All participants received study information and signed a written informed consent form in their preferred language (English or Spanish) after their questions were answered and before any research activities began. The consent form included the following information: (1) the primary data collection (surveys and interviews) would be used to inform intervention design; (2) interview recordings and transcripts would be deidentified and stored separately from study documents containing participants’ identifiable information on a password-protected, encrypted cloud drive to ensure data security and confidentiality; and (3) each participant would receive a US $80 gift card upon completing both the survey and the interview session.

## Results

### Stage 1: Exploration of Ostomy Care Experiences and Intervention Preference

#### Participant Characteristics

Data collection and analysis continued until no new themes related to care needs and ostomy experiences emerged during the interviews. The research team successfully enrolled 9 patients and 5 caregivers, reaching data saturation with a total of 14 participants. These participants had a mean age of 58.9 (SD 13.01, range: 37‐79) years. The mean CCI score was 3.21 (SD 1.86, range 0‐6), indicating a moderate comorbidity burden. The mean eHEALS score was 29.71 (SD 6.62, range 18‐40). Most participants (n=10) reported a high school diploma or General Educational Development equivalent as their highest level of education, while 2 participants had completed up to the seventh grade, and 2 participants held a bachelor’s degree. The patients and caregivers reported an annual income between US $10,000 and US $90,000. Most patients had their ostomy surgery 3‐4 years before the interview.

#### Thematic Analysis Results

##### Hispanic Patients and Caregivers’ Experiences With Ostomy Care

As outlined in Table 2, the thematic analysis identified three major themes and associated subthemes among Hispanic patients with cancer and caregivers: (1) perceptions of ostomy and its impact on their lives, (2) experiences in seeking support, and (3) challenges faced following discharge from the hospital.

##### Perceptions of Ostomy and Its Impacts

Patients and caregivers frequently expressed fear and negative perceptions regarding ostomies and used terms like “ugly” and “shocking” to describe them (refer to “Emotion” in Table 2). On the other hand, the subtheme of necessity reflected a gradual shift in ostomy perception as patients and caregivers began to acknowledge the life-saving function of an ostomy and were able to accept and live with it (refer to “Necessity” in Table 2).

##### Support Seeking

Support emerged as a significant theme from the interviews, with 4 sources identified, such as health care provider support, family and caregiver support, peer support, and self-care. Many participants reported limited access to resources with sufficient knowledge for changing ostomy bags and teaching others to perform the task (refer to “Health care provider” in Table 2). As shown in the “Family and caregiver” section in Table 2, patients with CCI scores ranging from 4 to 6 emphasized the crucial role of family support. Some indicated that family members often act as backups for one another when providing care. While some patients do not formally recognize their family members as caregivers, they still rely on them for ostomy bag changes. Additionally, patients expressed a desire to get help from people who have had similar experiences (ie, “Peer support” in Table 2. Conversely, patients with relatively low CCI scores highlighted the importance of self-reliance during postsurgical home management (refer to “Self-care” in Table 2).

##### Challenges Post Surgery

Patients faced significant physical and emotional difficulties after ostomy surgery. Many needed to continue cancer treatment, such as radiation and chemotherapy, which further complicated their daily ostomy care (refer to “Radiation side effects” and “Chemotherapy side effects” in Table 2). Side effects of these treatments, such as fatigue and diarrhea, made it harder for patients to manage bag changes, thereby increasing the risk of leaks. Additionally, treatments made patients’ skin more sensitive and prone to irritation (refer to “Skin irritation” in Table 2). As shown in the “Leakage” section in Table 2, leakage challenges were associated with obesity, which is a common issue in the Hispanic community. Leakage not only caused feelings of helplessness but also led to self-isolation. Many patients reported feeling more secure and comfortable staying at home (refer to “Self-isolation” in Table 2). Notably, caregivers reported high levels of stress and depression while caring for patients with ostomy cancer (refer to “Caregiver stress and emotion” in Table 2).

### Preference for the PACE Ostomy Care Intervention Program

#### “insert subheading”

Overall, participants felt excited about the existence of a program like PRISMS and expressed satisfaction with the translated PRISMS-Spanish materials provided during the interviews. They also shared their preferences regarding intervention delivery timing, devices commonly used in information searching (mobile phones, desktops, etc), and supporting resources.

#### Importance of Preferred Language and Multimedia Delivery

Participants reported a preference for multimedia, including text documents, audio, and videos, when presented with intervention materials. They found practical tips and tricks extremely useful, especially regarding the optimal timing for changing ostomy appliances, barrier rings, and support belts. Although some tips had been mentioned by their health care team before, participants appreciated the tips as valuable reminders (refer to “Content and delivery format” in Table 3). Participants with low eHEALS scores (<26) were more likely to be impacted by language barriers when accessing health care resources. This is supported by the “Language” (refer to “Language” in Table 3) subtheme, where participants expressed a preference for Spanish-translated materials.

#### Need for Early Introduction, Multimodal Materials Delivery and Peer and Family Support

Participants expressed their preference for the intervention delivery platform, including the timing and support they would like to receive. Many participants were proficient with computers and mobile devices, but some suggested that providing printed handouts with pictures would also be helpful (refer to “Delivery platform” in Table 3). Participants also stated that information about ostomy care should be provided at the preoperative appointment or, at the latest, during hospitalization, although most patients were sent home with discharge paperwork after having a nurse demonstrate a bag change (refer to “Delivery timing” in Table 3). Feedback also emphasized the importance of training multiple support people in changing an ostomy bag, depending on family dynamics (refer to “Family and peer support” in Table 3). These comments further illustrated that the program should be accessible to all relevant family members.

### Stage 2 Design of the Web-Based PACE Intervention

Based on the feedback in stage 1, we developed the culturally adapted PRISMS-Spanish intervention, renamed as PACE. In this section, we focus on introducing the design of the PACE model, guided by affinity diagramming and user engagement strategies informed by the PSD model.

#### Content and Cultural Adaptation

Based on the interview findings and thematic analysis, we primarily focused on culturally adapting and customizing the existing PRISMS/PRISMS-Spanish program to develop the PACE intervention for Hispanic patients with cancer and their family members. PACE retained core contents from the PRISMS, including text and demonstrative videos on ostomy care, providing information on managing common complications (eg, dehydration and skin issues), performing safe physical activity, and preventing falls. Additionally, we introduced a new preoperation checklist to help patients and their families prepare thoroughly. In response to participants’ preferences, we maintained the PRISMS-Spanish for information delivery using multimedia formats, including audio, video, and text. For instance, a step-by-step video demonstration of the crusting technique and a text listing situations in which patients should contact a doctor post surgery were included in PACE. To further enhance user experience, audio, and video were adapted to accommodate preferences for speaking speeds, tone, and captioning.

New culturally adaptive elements were added to engage Hispanic users by incorporating familiar visuals and culturally resonant content. For example, images of traditional Hispanic dishes, such as empanadas and ceviche, were included to promote healthy eating. Culturally significant colors like orange and blue were used to evoke a sense of energy and warmth. These cultural adaptations aimed to develop an inclusive platform for PACE that fosters connection and engagement within the Hispanic community, optimizing support for patients with cancer and caregivers.

#### Affinity Diagram of the PACE Website

**Figure 1.**
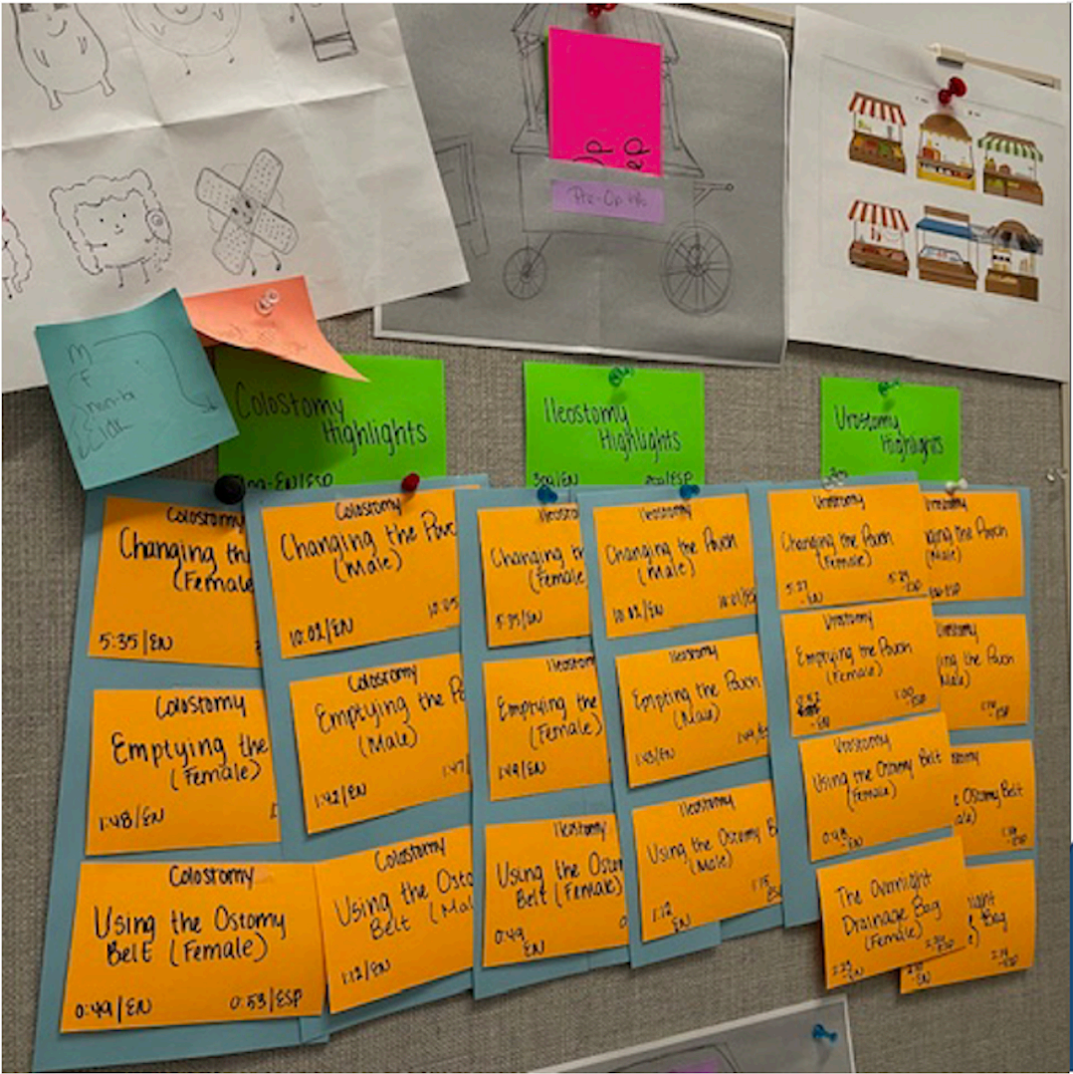
Affinity diagram of the PACE (Programa de AutoCuidado de Estoma) website.

An affinity diagram was created to organize the content in a logical and user-friendly layout (Figure 1). Each piece of material, whether text, videos, or audio files, was represented as a color-coded data point and written on sticky notes, which were mapped into meaningful sections informed by interview feedback on delivery timing. This process aided in designing a user experience that guides users through the entire journey of ostomy care, from preoperative preparation to postsurgery self-management at home, with specific adaptations for the Hispanic population.

**Figure 2.**
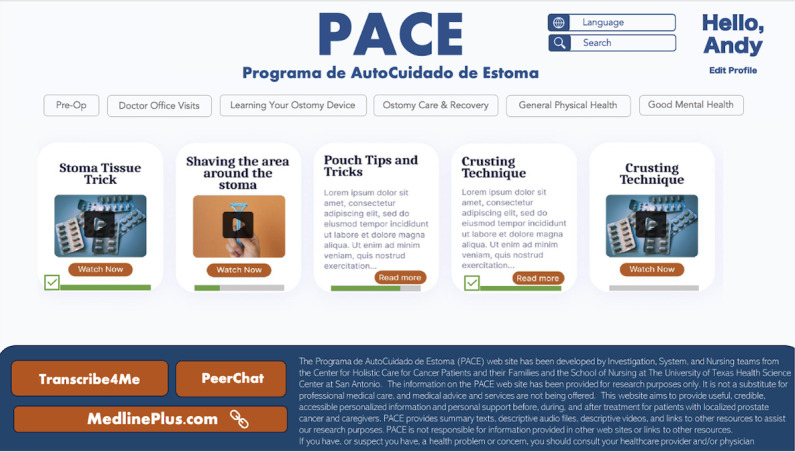
Home page of the PACE (Programa de AutoCuidado de Estoma) website.

Similar to our PRISMS intervention, our team identified 6 main sections for the PACE website: “Pre-Op,” “Doctor Office Visits,” “Learning Your Ostomy Device,” “Ostomy Care & Recovery,” “General Physical Health,” and “Good Mental Health” (Figure 2). “Pre-Op” offers comprehensive preoperative preparation, including a printable medication list template. “Doctor Office Visits” provides checklists for health care appointments, listing symptoms and test results to track. “Learning Your Ostomy Device” delivers essential knowledge on managing an ostomy, including changing, emptying, and understanding different types of bags and related equipment. “Ostomy Care & Recovery” addresses skin irritations, chemotherapy side effects, and common complications. “General Physical Health” offers recommendations for physical activities and tips on nutrition and dressing for fitness. Finally, “Good Mental Health” provides strategies for maintaining mental well-being for both patients and caregivers.

#### Persuasive Elements

##### Overview

After analyzing the interview data and designing the website layout, the research team (LS, AV, JL, LRR, and XW) conducted an expert evaluation leading to the integration of 7 persuasive principles of the PSD Model into the website design, as detailed in Table 4.

**Table 4.** Persuasive elements in PACE (Programa de AutoCuidado de Estoma).

PSD^a^ categories and selected principles for PACE^b^ design	Application of PSD principles
Primary task support	
Tailoring	Users receive tailored task recommendations based on their group (Patient/Caregiver and family member).
Self-monitoring	Users can track their progress on each task.
Dialogue support	
Rewards	Users earn avatars as rewards for progress in learning sections.
Reminders	Users receive email reminders to log in to the PACE website regularly.
Credibility support	
Trustworthiness	The PACE website offers truthful, fair, and unbiased information through various media, including videos, audio, text, and images.
Real-world feel	Team members from the research and development teams are introduced on the login page.
Social support	
Social learning	An online forum is available in PACE, allowing users to write comments, ask questions, or share experiences anonymously.

a PSD: Persuasive Systems Design.

b PACE: Programa de AutoCuidado de Estoma.

##### Primary Task Support

We incorporated tailoring and self-monitoring principles. Thematic analysis revealed that many participants desired multiple family members to be trained in ostomy care. To address this, we applied the tailoring principle by enabling role selection and rendering customized content. During registration, users could choose roles (patient, caregiver, or other support) and have the flexibility to change roles later in the user profile settings. The content and layout of the website were rearranged according to user roles, enhancing the user experience. For UI design, our team decided to use a card layout (clickable containers with images and a brief content summary) with a progress bar at the bottom (Figure 2B) by displaying completed tasks and allowing users to resume where they left off to support self-monitoring. Users could track their progress in completing recommended videos and audios on their profile page.

##### Dialogue Support

To reduce fear and stress and encourage engagement, we established a reward mechanism where users could unlock new cartoon ostomy avatars and decorations after engaging with content. Additionally, email reminders were also set to prompt participants to revisit the website.

##### Credibility Support

To ensure the PACE program’s credibility, we applied the principles of trustworthiness and real-world feel. We presented truthful, fair, and unbiased information through videos, text, and images based on user preferences. The information was developed based on scientific evidence and clinical guidelines with guidance from health care professionals. Videos were recorded by a certified Wound and Ostomy Care Nurse from the University of North Carolina at Chapel Hill. Additionally, a detailed introduction of the research team was also displayed on the login page for reference.

##### Social Support

The social learning principle was facilitated through an online forum, “Peer Support” (Figure 2). After logging in to the PACE website, patients and their family members could join support groups and seek advice. They could post, comment, and bookmark discussions, creating a space for the Hispanic community to not only exchange ostomy care knowledge but also provide emotional support to those community members in need, fostering a sense of belonging.

## Discussion

### Principal Findings

The main findings of this study revealed that Hispanic patients with cancer and caregivers experienced significant emotional distress and faced multiple challenges related to ostomy care management post surgery. Participants emphasized the need for culturally tailored interventions and expressed preferences for bilingual multimedia content, early delivery of the intervention, and the inclusion of family and peer support in ostomy care management. Drawing on these insights and feedback, our multidisciplinary team culturally adapted the PRISMS-Spanish and designed and developed the bilingual PACE intervention.

First, participants in our study highlighted a strong preference for content in Spanish alongside English, aligning with existing literature that emphasizes the importance of bilingual support in efficiently overcoming linguistic barriers [32]. Participants who completed the eHEALS survey in Spanish and scored below 26 reported difficulties reading English-language materials and expressed a strong preference for Spanish-translated content. The limited availability of Spanish-language digital resources likely limited their opportunity to access online health information, contributing to lower confidence in using digital health tools and lower self-reported digital health literacy. These findings illustrate the importance of providing bilingual digital health intervention. Thus, we developed the bilingual PACE program by integrating the original PRISMS and the PRISMS-Spanish materials, ensuring cultural and contextual relevance for Hispanic patients and caregivers. Additionally, the bilingual PACE intervention was designed to facilitate family involvement in ostomy care, particularly among Hispanic children and younger generations who are fluent in English and more acculturated to American culture [33,34]. To accommodate participants’ preferences and address linguistic barriers, the bilingual platform includes Hispanic dubbing for audio and videos, in addition to text and graphics, which were recorded at appropriate speaking speeds in both English and Spanish, accommodating both patients and their family members who may not speak Spanish or English but still act as caregivers. Recent findings from the Pew Research Center indicate that approximately 65% of third- or later-generation Latinos report limited proficiency in Spanish [35]. By incorporating a bilingual focus, PACE fosters engagement with the entire family, regardless of language proficiency. Our study participants expressed satisfaction with the translation and bilingual design during interviews, highlighting its effectiveness in meeting their needs.

Second, the perceptions of our study participants also played a key role in shaping the delivery format of the PACE intervention. Hispanic patients and caregivers expressed a strong preference for content visualization through a multimedia platform, aligning with the findings of Joshi et al [36] in which Hispanic participants find multimedia programs easy to navigate and effective for obtaining relevant health information. In response, PACE delivers content in various formats, including video, audio, images, and text, to cater to diverse learning preferences. To further enhance engagement, we incorporated culturally relevant elements, such as images of Hispanic foods and vibrant colors like blue and orange, which resonate with the community. Additionally, based on participants’ preferences and feedback, similar to the original PRISMS, PACE was designed to be accessible across different devices, including personal computers, mobile phones, and tablets.

Third, participants’ feedback affirmed the comprehensive content of the original PRISMS and guided the design of PACE to improve QOL for patients and caregivers. Participants shared their experiences living with an ostomy, often expressing feelings of embarrassment and body image issues following surgery. By increasing knowledge and familiarity, PACE helps Hispanic patients and caregivers integrate ostomy care into their daily lives with greater ease and positivity.

Additionally, our study revealed that family members of Hispanic cancer survivors are often expected to provide emotional support, assist with daily living, and manage overall health care [37]. This cultural expectation includes providing comprehensive information about the illness [38], treatment details, anticipated side effects, hands-on care skills [39,40], and strategies for stress reduction [41,42]. However, these informational needs are often unmet [41-44]. To fill this gap, PACE delivers tailored information for both patients and their caregivers and family members, enhancing their knowledge and skills in ostomy care. This approach not only enables them to provide better support to patients but also helps maintain their own QOL.

Finally, and most importantly, we used PSD principles to enhance user engagement in the web-based PACE interventions. A systematic review [45] demonstrates that PSD can significantly enhance adherence to web-based interventions, particularly those targeting chronic conditions and lifestyle changes [46,47]. This is especially relevant for patients with cancer living with ostomies, who face substantial changes in their health, lifestyle, behaviors, and daily routines [48]. To promote effective self-management, patients and their families must consistently engage with web-based interventions [49]. However, a notable challenge is that Hispanics are reportedly less likely to use health-related applications compared to other racial groups [50]. Moreover, the absence of user input during program development can further limit the success of these interventions [51]. In this study, we applied PSD principles in the design of the PACE intervention to enhance user engagement and ostomy care self-management. Building on the features of the original PRISMS intervention and incorporating participant feedback, we implemented key PSD principles to enhance engagement. Findings from this study are consistent with prior research [52-54], demonstrating that integrating persuasive principles into intervention design can significantly improve user engagement and support the long-term effectiveness of web-based platforms. The following are examples of how we integrate PSD principles into the design of PACE.

1. Self-monitoring: Participants were encouraged to track their progress in learning ostomy care, increasing awareness, and reinforcing skill development.
2. Rewards and reminders: These features were incorporated to foster continuous use of the program, promoting the acquisition of strong ostomy care skills [55].
3. Trustworthiness and readability: The PACE intervention ensured information was truthful, fair, and unbiased, building trust with users through a user-friendly interface.
4. Social learning: An online forum was integrated, enabling peer support and communication within the PACE intervention.

### Strengths and Limitations

To our knowledge, this is the first study to apply affinity diagramming and PSD principles in the design of an ostomy care intervention. Given the limited access to high-quality online health resources among the Hispanic population [56] and building on evidence that Spanish-language, tailored health information in Spanish improves reach and effectiveness in this underserved group [57], our multidisciplinary team intentionally incorporated bilingual and culturally sensitive features to enhance patient engagement and family involvement in ostomy care. During cancer survivorship, ostomy creation as part of cancer treatment is a life-changing event. Traditional education, typically delivered through brief in-hospital demonstrations, can be overwhelming for patients recovering from surgery and coping with changes in physical function and body image. PACE addresses this gap by providing accessible, bilingual resources designed to meet the cultural and informational needs of Hispanic families. By supporting both patients and caregivers during the critical care transition from professional hospital care to self-management at home, PACE aims to improve health outcomes and support their caregivers’ well-being.

However, the following limitations warrant further exploration. First, this study lacked participation from family members other than the first-line caregivers in the interviews. Participants emphasized the importance of involving additional family members in ostomy care. Future studies should address this gap by including a broader range of family members of Hispanic patients with cancer to better capture their diverse experiences and needs. Second, participants’ educational attainment was at the high school level or above, which may limit the generalizability of the findings, given that a large proportion of Hispanic adults—particularly older or foreign-born individuals—have not completed high school [58]. Future research should prioritize recruiting individuals with lower educational attainment to rigorously design and evaluate digital interventions like PACE. Third, most patients in this study were more than 1 year post ostomy surgery, so their insights may differ from those who are newly diagnosed or recently discharged and still adjusting to life with an ostomy. We intentionally recruited this population because they had already navigated the postostomy survivorship journey and were likely to be more motivated, engaged, and interested in accessing online information compared with those who are fresh out of ostomy-creation surgery. While their reflection may be subject to recall bias, we encouraged them to draw on their lived experiences. Their insights were valuable in informing an intervention design that is potentially feasible and acceptable for individuals in the early stages of recovery. However, future studies should evaluate the acceptability, usability, feasibility, and effects of the intervention among patients and caregivers managing newly created ostomies to ensure its relevance and applicability across various stages of recovery.

Fourth, this study used descriptive statistics to analyze the quantitative data, which may limit the ability to draw statistical inferences about relationships between variables, such as age, education, digital literacy, comorbidities, and intervention preferences. Future studies with larger sample sizes need to incorporate statistical methods (eg, regression analysis or causal inference techniques) to examine associations between participant characteristics and engagement in digital health interventions. Finally, this study reported percent agreement to assess coding consistency, given the iterative coding approach and small sample size. Future work should consider applying more robust methods, such as Cohen κ, to evaluate interrater reliability in large-scale studies.

Despite these limitations, the findings support the cultural adaptation of the English version of the PRISMS intervention into a user-friendly bilingual PACE web-based program for Hispanic patients and families, a population often underrepresented in clinical intervention research. Future studies should recruit participants with diverse backgrounds to evaluate the PACE intervention in other underserved communities, focusing on improving health outcomes and addressing health disparities.

### Research and Clinical Implications

The PACE web-based program addresses critical challenges in ostomy care, including skin irritation, leakage, treatment side effects, and social and emotional stress with the goal of improving health outcomes for patients and caregivers. PACE provides adaptable techniques for different stages of recovery, standardizes patient and family education, and supports self-management in the hospital and at home. Its scalability and accessibility are particularly valuable for individuals in remote areas, such as those in rural Texas. By integrating findings from the PRISMS pilot feasibility study and current research, PACE is positioned for testing in a sufficiently powered clinical trial to evaluate its efficacy and effectiveness. Making the program accessible in hospital rooms could further enhance its impact on patient education and care transitions.

### Conclusions

Our findings revealed the experiences of Hispanic patients and caregivers managing ostomy care for cancer treatment, including their support-seeking behaviors, postdischarge challenges, and preferences related to language, timing of intervention delivery, digital platforms, and the inclusion of family and peer support. Informed by these insights, we culturally adapted the PRISMS-Spanish program and applied PSD principles to design the bilingual, web-based PACE intervention. This tailored approach aims to enhance patient and caregiver engagement, strengthen their ostomy care skills, and ultimately improve their health outcomes. Future research will include a pilot testing for usability and feasibility of the PACE intervention among larger and more diverse Hispanic populations.

## Supplementary material

10.2196/70354 Checklist 1 Mixed Methods Reporting in Rehabilitation and Health Sciences (MMR-RHS).

## Abbreviations

CCI: Charlson Comorbidity Index
eHEALS: eHealth literacy scale
IRB: Institutional Review Board
PACE: Programa de AutoCuidado de Estoma
PRISMS: Patient-Reported Outcomes-Informed Symptom Management System
PSD: Persuasive Systems Design
QOL: quality of life
REDCap: Research Electronic Data Capture
UI: user interface
UTHSA: University of Texas Health San Antonio

## References

[1] Krouse RS, Grant M, McCorkle R (2016). A chronic care ostomy self-management program for cancer survivors. Psychooncology.

[2] Yu H, Ryu E (2024). Colorectal cancer survivors’ inner strength, multiple identities, and quality of life by gender and ostomy presence: a cross-sectional study. Korean J Adult Nurs.

[3] Victorson D, Banas J, Smith J (2014). eSalud: designing and implementing culturally competent ehealth research with latino patient populations. Am J Public Health.

[4] Costas-Muñiz R, Hunter-Hernández M, Garduño-Ortega O, Morales-Cruz J, Gany F (2017). Ethnic differences in psychosocial service use among non-Latina white and Latina breast cancer survivors. J Psychosoc Oncol.

[5] Creamer J Inequalities persist despite decline in poverty for all major race and hispanic origin groups. United States Census Bureau.

[6] Viale PH (2020). The American Cancer Society’s Facts & Figures: 2020 Edition. J Adv Pract Oncol.

[7] Duran M (2012). Rural Hispanic health care utilization. Online J Rural Nurs Health Care.

[8] Sehar U, Rawat P, Choudhury M (2023). Comprehensive understanding of Hispanic caregivers: focus on innovative methods and validations. J Alzheimers Dis Rep.

[9] Sharp SP, Ata A, Chismark AD (2020). Racial disparities after stoma construction in colorectal surgery. Colorectal Dis.

[10] Kronenfeld JP, Graves KD, Penedo FJ, Yanez B (2021). Overcoming disparities in cancer: a need for meaningful reform for Hispanic and Latino cancer survivors. Oncologist.

[11] Bisht J, Rawat P, Sehar U, Reddy PH (2023). Caregivers with cancer patients: focus on Hispanics. Cancers (Basel).

[12] Golpazir-Sorkheh A, Ghaderi T, Mahmoudi S, Moradi K, Jalali A (2022). Family-centered interventions and quality of life of clients with ostomy. Nurs Res Pract.

[13] Heydari A, Manzari ZS, Pouresmail Z (2023). Nursing intervention for quality of life in patients with ostomy: a systematic review. Iran J Nurs Midwifery Res.

[14] Solomon M, Wagner SL, Goes J (2012). Effects of a web-based intervention for adults with chronic conditions on patient activation: online randomized controlled trial. J Med Internet Res.

[15] Wantland DJ, Portillo CJ, Holzemer WL, Slaughter R, McGhee EM (2004). The effectiveness of web-based vs. non-web-based interventions: a meta-analysis of behavioral change outcomes. J Med Internet Res.

[16] Larsen B, Marcus B, Pekmezi D, Hartman S, Gilmer T (2017). A web-based physical activity intervention for Spanish-speaking Latinas: a costs and cost-effectiveness analysis. J Med Internet Res.

[17] Gans KM, Dulin A, Palomo V (2021). A tailored web- and text-based intervention to increase physical activity for Latino men: protocol for a randomized controlled feasibility trial. JMIR Res Protoc.

[18] Fortmann AL, Savin KL, Clark TL, Philis-Tsimikas A, Gallo LC (2019). Innovative diabetes interventions in the U.S. hispanic population. Diabetes Spectr.

[19] Xu S, Tan X, Ma C (2023). An eHealth symptom and complication management program for cancer patients with newly created ostomies and their caregivers (Alliance): a pilot feasibility randomized trial. BMC Cancer.

[20] Terry G, Hayfield N, Clarke V, Braun V The SAGE Handbook of Qualitative Research in Psychology.

[21] Oinas-Kukkonen H, Harjumaa M (2009). Persuasive systems design: key issues, process model, and system features. Commun Assoc Inf Syst.

[22] Guan V, Zhou C, Wan H (2023). A novel mobile app for personalized dietary advice leveraging persuasive technology, computer vision, and cloud computing: development and usability study. JMIR Form Res.

[23] Idrees AR, Kraft R, Mutter A, Baumeister H, Reichert M, Pryss R (2024). Persuasive technologies design for mental and behavioral health platforms: a scoping literature review. PLOS Digit Health.

[24] (2023). ACS demographic and housing estimates. United States Census Bureau.

[25] Folstein MF, Folstein SE, McHugh PR (1975). “Mini-mental state”. A practical method for grading the cognitive state of patients for the clinician. J Psychiatr Res.

[26] Charlson ME, Carrozzino D, Guidi J, Patierno C (2022). Charlson comorbidity index: a critical review of clinimetric properties. Psychother Psychosom.

[27] Norman CD, Skinner HA (2006). eHEALS: The eHealth Literacy Scale. J Med Internet Res.

[28] Guest G, Bunce A, Johnson L (2006). How many interviews are enough?: an experiment with data saturation and variability. Field methods.

[29] Miles MB, Huberman AM (1994). Qualitative Data Analysis: An Expanded Sourcebook.

[30] Harboe G, Huang EM Real-world affinity diagramming practices: bridging the paper-digital gap. https://dl.acm.org/doi/10.1145/2702123.2702561.

[31] Asbjørnsen RA, Smedsrød ML, Solberg Nes L (2019). Persuasive system design principles and behavior change techniques to stimulate motivation and adherence in electronic health interventions to support weight loss maintenance: scoping review. J Med Internet Res.

[32] Banas JR, Victorson D, Gutierrez S, Cordero E, Guitleman J, Haas N (2017). Developing a peer-to-peer mHealth application to connect Hispanic cancer patients. J Canc Educ.

[33] Toppelberg CO, Collins BA (2010). Language, culture, and adaptation in immigrant children. Child Adolesc Psychiatr Clin N Am.

[34] Hakimzadeh S, Cohn DV (2007). English usage among hispanics in the united states. https://www.pewresearch.org/race-and-ethnicity/2007/11/29/english-usage-among-hispanics-in-the-united-states/.

[35] Mora L, Lopez MH (2023). Latinos’ views of and experiences with the Spanish language. https://www.pewresearch.org/race-and-ethnicity/2023/09/20/latinos-views-of-and-experiences-with-the-spanish-language/.

[36] Joshi A, Wilhelm S, Aguirre T, Trout K, Amadi C (2013). An interactive, bilingual touch screen program to promote breastfeeding among Hispanic rural women: usability study. JMIR Res Protoc.

[37] Enriquez M (2019). *Hispanic Health Care International*: meeting the needs of our readers. Hisp Health Care Int.

[38] Badger TA, Sikorskii A, Segrin C (2019). Contextual and cultural influences on caregivers of Hispanic cancer survivors. Semin Oncol Nurs.

[39] Brown CG (2010). A guide to oncology symptom management. Oncology Nursing Society.

[40] Lopez V, Copp G, Molassiotis A (2012). Male caregivers of patients with breast and gynecologic cancer: experiences from caring for their spouses and partners. Cancer Nurs.

[41] Loke AY, Liu CFF, Szeto Y (2003). The difficulties faced by informal caregivers of patients with terminal cancer in Hong Kong and the available social support. Cancer Nurs.

[42] Sklenarova H, Krümpelmann A, Haun MW (2015). When do we need to care about the caregiver? Supportive care needs, anxiety, and depression among informal caregivers of patients with cancer and cancer survivors. Cancer.

[43] Chua GP, Ng QS, Tan HK, Ong WS (2020). Caregivers of cancer patients: what are their information-seeking behaviours and resource preferences?. Ecancermedicalscience.

[44] van Ryn M, Sanders S, Kahn K (2011). Objective burden, resources, and other stressors among informal cancer caregivers: a hidden quality issue?. Psychooncology.

[45] Kelders SM, Kok RN, Ossebaard HC, Van Gemert-Pijnen JEWC (2012). Persuasive system design does matter: a systematic review of adherence to web-based interventions. J Med Internet Res.

[46] Alpay L, Doms R, Bijwaard H (2019). Embedding persuasive design for self-health management systems in Dutch healthcare informatics education: application of a theory-based method. Health Informatics J.

[47] Asbjørnsen RA, Hjelmesæth J, Smedsrød ML (2022). Combining persuasive system design principles and behavior change techniques in digital interventions supporting long-term weight loss maintenance: design and development of eCHANGE. JMIR Hum Factors.

[48] Ercolano E, Grant M, McCorkle R (2016). Applying the chronic care model to support ostomy self-management: implications for oncology nursing practice. Clin J Oncol Nurs.

[49] Moulaei K, Iranmanesh E, Ahmadian L (2023). The impact of health technologies on ostomy care: a systematic review of health technologies impact on ostomy care. J Wound Ostomy Continence Nurs.

[50] Chae J (2018). A comprehensive profile of those who have health-related apps. Health Educ Behav.

[51] Alkhaldi G, Hamilton FL, Lau R, Webster R, Michie S, Murray E (2016). The effectiveness of prompts to promote engagement with digital interventions: a systematic review. J Med Internet Res.

[52] Sporrel K, Nibbeling N, Wang S, Ettema D, Simons M (2021). Unraveling mobile health exercise interventions for adults: scoping review on the implementations and designs of persuasive strategies. JMIR Mhealth Uhealth.

[53] Asbjørnsen RA, Wentzel J, Smedsrød ML (2020). Identifying persuasive design principles and behavior change techniques supporting end user values and needs in eHealth interventions for long-term weight loss maintenance: qualitative study. J Med Internet Res.

[54] Abdullahi AM, Orji R, Rabiu AM, Kawu AA (2020). Personality and subjective well-being: towards personalized persuasive interventions for health and well-being. Online J Public Health Inform.

[55] Wang Y, Mo DY, Ma HL (2023). Perception of time in the online product customization process. Ind Manag Data Syst.

[56] Chavarria EA, Christy SM, Feng H (2022). Online health information seeking and eHealth literacy among Spanish language–dominant Latino adults receiving care in a community clinic: Secondary analysis of pilot randomized controlled trial data. JMIR Form Res.

[57] Linke SE, Dunsiger SI, Gans KM (2019). Association between physical activity intervention website use and physical activity levels among Spanish-speaking Latinas: randomized controlled trial. J Med Internet Res.

[58] (2020). 2020 profile of Hispanic Americans aged 65 and older.

